# Serum Interleukin-6 in Patients with Burning Mouth Syndrome and Relationship with Depression and Perceived Pain

**DOI:** 10.1155/2007/45327

**Published:** 2007-04-22

**Authors:** Qianming Chen, Juan Xia, Mei Lin, Hongmei Zhou, Bingqi Li

**Affiliations:** ^1^Department of Oral Medicine, West China College of Stomatology, Sichuan University, Chengdu, Sichuan 610041, China; ^2^Department of Oral Medicine, Guanghua School of Stomatology, Sun Yat-Sen University, Guangzhou, Guangdong 510055, China

## Abstract

*Objective*. To examine alteration of serum interleukin-6 and its clinical significance in burning mouth syndrome (BMS) patients. *Methods*. 48 BMS patients and 31 healthy controls participated in the study. Serum interleukin-6 was measured by means of ELISA. Hamilton rating scale of depression (HRSD) and visual analogue scale (VAS) were used to quantitiate depressive status and pain levels of subjects, respectively. *Results*. 15 (31%) patients displayed substantial depressive symptoms (HRSD ≧ 16). HRSD scores of patients were significantly higher than controls and positively correlated to their VAS values (*P* = .002). Serum interleukin-6 in patients was much lower than controls and negatively correlated to their VAS values (*P* = .011). However, no significant relations were found between interleukin-6 and HRSD scores (*P* = .317). *Conclusions*. Serum interleukin-6 in patients with burning mouth syndrome is decreased and negatively correlated to chronic pain. Both psychological and neuropathic disorders might act as precipitating factors in BMS etiopathogenesis.

## 1. INTRODUCTION

Burning mouth syndrome (BMS) is a chronic condition characterized by intraoral burning pain in the absence of identifiable clinical abnormalities, affecting predominantly middle-aged women. Multiple oral sites may be involved and the anterior two-thirds and tip of tongue are most commonly affected. Besides the burning sensation, a variety of symptoms could be simultaneously present, such as xerostomia, dysgeusia, and psychological dysfunction [1]. To date, despite a large body of knowledge, BMS etiology remains largely enigmatic and presents challenge for both researchers and clinicians.

Neuropathic background in BMS etiopathogenesis has been underscored recently by more evidence [2]. Abnormal perception of intensity in the prepain range and of thermal stimuli raised trigeminal nerve sensitivity, alterations in neuronal transmission, and disturbances of the mucosal neurovascular microcirculatory system have been documented in BMS patients [3–5]. All those approve the neuropathic view on BMS.

Intriguingly, interleukins as potential neuromodulators/neurotransmitters are providing increasingly impressive database for their roles in the nociceptive processing which lead to neuropathic pain and/or hyperalgesia [6]. Interleukin-6 (IL-6), one of the neuropoietic cytokines and attracting most attentions, could play various roles in nervous system including glia proliferation, neuronal survival and differentiation, axonal regeneration, and proinflammatory activities [7–9]. Considering the neuropathic basis for BMS and the potential neurological actions of IL-6, it seems interesting to explore association between IL-6 and BMS. Early studies showed conflicting data of salivary IL-6 in BMS patients when compared to the healthy controls [10, 11]; nevertheless, how about serum IL-6 and its clinical significance?

Therefore, in the present study, serum IL-6 was measured by enzyme linked immunosorbent assay (ELISA) kits and its relationship with clinical symptoms, such as depressive symptoms and pain levels, was also determined in BMS patients.

## 2. SUBJECTS AND METHODS

### 2.1. Patients and healthy controls

This was a case control study. Patients, meeting diagnostic criteria of BMS, were recruited from the Department of Oral Medicine, West China College of Stomatology, Sichuan University. The including criteria were based on a chief complaint of oral pain on the tongue. Furthermore, absence of clinical signs was necessary and was verified in all cases by two professional dentists with good interexaminer consistency. Controls came from the subjects who voluntarily resorted to our hospital for healthy examinations. Individuals would be excluded if they suffered from other local or systemic disease, took immunodepressant or immunopotentiating drugs during the previous three months. Data including age, gender, medical history, drug history, and clinical symptoms were recorded in detail. Informed consent to participate in this study was obtained from all subjects after both verbal and written study examinations. The protocol was submitted and approved by our Institutional Review Board (IRB).

### 2.2. Clinical assessment

Two clinical parameters, pain and psychological status, were evaluated in subjects if appropriate. The intensity of pain was measured by using visual analogue scale (VAS). Patients were instructed to bisect a 10 cm line from 0 (no pain) to 10 (extreme pain) at a point appropriate to quantify their pain discomfort. Meanwhile, the 17-item Hamilton rating scale of depression (HRSD) was used to measure depressive status of all subjects [12]. According to the quantitative severity of depressive symptoms, patients were divided into two subgroups, one with an HRSD ≧ 16 (BMS + D, indicating substantial depressive symptoms) and the other with an HRSD < 16 (BMS − D) [13].

### 2.3. IL-6 determination

Blood samples were obtained from all subjects after an overnight fast at room temperature between 9:00 am and 11:00 am. By using commercially available ELISA kits (R&D Systems, Minneapolis, USA), serum IL-6 concentrations were determined in all samples according to the manufacturer's instructions. The reproducibility of this examination was confirmed by processing all samples twice.

### 2.4. Statistical analysis

The quantitative data were going through the normal distribution test. Normal distribution data were analyzed by means of analysis of variance (ANOVA). The qualitative data were subject to the chi-square analysis. Spearman's Rho correlation coefficient was used to analyze the relationship of IL-6 levels with VAS values and HRSD scores. Probability value of *P* < .05 (two-sided) was accepted as statistically significant for all statistical tests carried out in the present study by using SPSS^®^ version 10.0 software (SPSS Inc., Chicago, IL, USA).

## 3. RESULTS

### 3.1. Subject characteristics

A total of 79 subjects participated in this study, consisting of 48 BMS patients and 31 healthy controls. The patient group (age range 26–80 years, mean 49 ± 12 years) comprised 39 (81.25%) females and 9 (18.75%) males, with female : male ratios of 4 : 1. In control group there were 25 females and 6 males, and the mean age was 49 ± 11 years ranging from 30 to 61 years. No significant differences were found in age (*P* = .897) and gender ratios (*P* = .947) between BMS patients and healthy controls. Of the 48 BMS patients interviewed, 69.23% (*n* = 27) were postmenopausal women. Moreover, 25 (52%) patients showed oral dryness of different degrees and 6 (13%) patients complained of poor tastes.

The VAS value as recorded on Table 1 was remarkably higher (*P* < .05) in BMS + D subgroup compared with BMS − D subgroup, averaging 4.4 in the whole BMS patients. It should be noted that 15 (31%) patients displayed substantial depressive symptoms (HRSD ≧ 16) with a mean value of 20.1, while the remaining 69% (*n* = 33, HRSD < 16) showed a mean of 6.4. However, HRSD scores in the healthy controls were only 2.2 on average, which were significantly lower than both subgroups (*P* < .05).

### 3.2. Serum IL-6 and relationship with clinical parameters

Table 1 presented that serum IL-6 was significantly lower in patient group than the control (*P* < .05), whereas, no significant difference (*P* = .548) was observed between the two subgroups of BMS.

Correlation analysis demonstrated that serum IL-6 in BMS patients was negatively correlated to their VAS values (*r* = −.362, *P* = .011). Moreover, positive relationship was found between HRSD and VAS scores (*r* = .437, *P* = .002). However, serum IL-6 and HRSD scores were independent from each other without any association (*r* = −.132, *P* = .317).

## 4. DISCUSSION

BMS is a relatively common disease that can severely affect life quality of patient. Although its etiology remains elusive, depressive disorder is indicated as a precipitating factor [14]. In agreement, our data demonstrated that 15 (31%) patients showed substantial depressive symptoms and the patient with higher HRSD score tended to experience more severe pain (*r* = .437, *P* = .002). Thus, the HRSD might be useful in evaluating depressive symptoms and subsequently guiding clinical treatment in BMS patients.

Not only psychological distress, but also alterations in the trigeminal nociceptive pathway at peripheral and/or central nervous system have been broadly concerned in BMS, which support the neuropathic basis of BMS. The lingual branch of the mandibular division of the trigeminal nerve provides afferent fibers to the anterior two-thirds of the tongue, as well as to the lingual gingiva and the floor of the mouth. BMS patients display a decreased density of unmyelinated nerve fibers within epithelium, which shows a trend toward negative correlation with duration of pain, but not severity of pain [2]. Similarly, in our study, serum IL-6 decreased significantly (*P* < .05) in BMS patients, which negatively correlated with pain levels (*P* = .011), but not depressive status. In other words, the lower serum IL-6 is, the more severe pain BMS patient tends to experience. Our results, together with the tight relationship of IL-6 with nervous system and neuropathic diseases [13, 15], lead to a suggestion that IL-6 might be involved in BMS etiopathogenesis, which meanwhile strengthens the view of neuropathic background in BMS.

Different outcomes of IL-6 have been documented in various neuropathic diseases, though decreased IL-6 in the peripheral blood was found in our study. Patients with persistent sciatic pain display elevated IL-6 levels in the blood [16] and following traumatic brain injury, increased IL-6 levels are found in the brain [17]. Otherwise, either unchanged [10] or elevated [11] salivary IL-6 levels in BMS patients have been reported. Until now, little is known about the reason for those different changes and their functional significance. In general, IL-6 could mediate immune-response in the nervous system in response to injury or disease, which has been suggested to be “a double-edged sword” [18]. Thus, IL-6 could exert either detrimental or beneficial effects on the nervous system, which perhaps depend on the timing and levels of IL-6 expression. Although the inflammation after spinal cord injury is traditionally regarded as being detrimental, when delivered intraperitoneally or subcutaneously, exogenous IL-6 is beneficial for axonal regeneration [9] and improves signs and electrophysiological evidence of nerve dysfunction associated with diabetes-related neuropathy in streptozotocin (STZ) rats [19]. On the contrary, IL-6 deficient mice exhibits lower nociceptive threshold to both mechanical and thermal stimuli, and thus is hyperalgesia in comparison to their wild-type controls [20]. From above, we speculate that the neuroprotective and/or neuroreparative function of IL-6 could be modulated by levels of IL-6. Then, decreased IL-6 levels might contribute to hyperalgesia for its attenuated neuroprotective effects, which subsequently provides a further nice explanation to our results. Briefly, neuroprotective effects of serum IL-6 on trigeminal nociceptive pathway through some unknown mechanisms might be weakened for lower IL-6 concentrations, which could aggravate hyperalgesia in BMS as another precipitating factor. Yet, this view is still let to be substantiated by further researches, such as IL-6 alterations at local mucosal tissues.

Taken together, these data highlighted the psychological and neuropathic background of BMS. The neurotrophic and/or neuroprotective actions of IL-6 on trigeminal nociceptive pathway might be alleviated in response to decreased serum IL-6 levels, which subsequently contribute to the chronic pain in BMS. Nevertheless, more proofs should be provided in order to confirm and further characterize IL-6 signaling pathway in BMS, which might shed lights to novel diagnostic and therapeutic strategies in future.

## Figures and Tables

**Table 1:** Serum IL-6 concentrations and VAS scores in healthy controls and BMS patients subdivided into those with (BMS + D) and without (BMS − D) an HRSD score ≧ 16.

Group	HRSD	IL-6 (pg/mL)	VAS

Control (*n* = 31)	2.2 ± 1.4	10.3 ± 0.5	—
BMS (*n* = 48)	10.6 ± 7.4	7.4 ± 1.5	4.4 ± 2.1
BMS − D (*n* = 33)	6.4 ± 3.3	7.5 ± 1.6	3.9 ± 1.9
BMS + D (*n* = 15)	20.1 ± 4.3	7.1 ± 1.4	5.6 ± 2.0
*F* *(df)	39.854(1/77)	102.769(1/77)	—
*P* * value	.000 < .05	.000 < .05	—
*F* **(df)	147.336(1/46)	.367(1/46)	7.411(1/46)
*P* ** value	.000 < .05	.548 > .05	.009 < .05

*All ANVOAs, showing the differences between healthy controls and BMS patients.

**All ANVOAs, showing the differences between BMS − D and BMS + D subgroups.
